# The Effect of Social Activities on the Life Satisfaction of Korean Older Adults

**DOI:** 10.1093/geroni/igaa057.1610

**Published:** 2020-12-16

**Authors:** Meeryoung Kim

**Affiliations:** Daegu University, Seoul, Republic of Korea

## Abstract

As life expectancy increases, older adults need to find ways to occupy their time for 20-30 years. For Korean older adults, social activities such as having relationships with others as well as, involvement in organizations and volunteer work, are important for their social identity. Social activities are one of the categories of Rowe and Kahn’s successful aging, this study examined the effect of having relationships, involvement in organizations and volunteering on the life satisfaction of older adults. This study used the 6th additional wave of the Korean Retirement and Income Study (2016). The target population was older adults (50~59, 60~74, 75+). The sample size was 1,921, 2,344 and 962 respectively. For data analysis, ANOVA and multiple regressions were used. The demographic variables were controlled. As for independent variables, having relationships, involvement in organizations, and volunteering were used. For the dependent variable, life satisfaction was used. Having relationships, involvement in organizations and volunteering were significantly different by age group. For each age group, the factors affecting life satisfaction differ. For the middle aged group, involvement in organizations and volunteering were significant factors affecting life satisfaction. For young-old adults, volunteering had the most significant effect on life satisfaction. Finally, for old-old adults, both having human relations and involvement in organizations were significant. These findings imply that social activities differ by age group. Also, the kind of activities affecting life satisfaction differ by age group. These findings imply that it is important for older adults to be involved in society, in various ways.

